# Lung cyst and multinodular thyroid goiter: Keys to DICER1 syndrome diagnosis in a 16‐year‐old female

**DOI:** 10.1002/ccr3.3098

**Published:** 2020-07-17

**Authors:** James D. Tutor, Stephen F. Miller, Hiba Al Zubeidi, Anthony Sheyn, Jie Zhang, Regan Williams, Rose B. McGee

**Affiliations:** ^1^ Department of Pediatrics University of Tennessee Health Science Center Memphis TN USA; ^2^ LeBonheur Children's Hospital Memphis TN USA; ^3^ Consulting Physician St. Jude Children's Research Hospital Memphis TN USA; ^4^ Department of Radiology University of Tennessee Health Science Center Memphis TN USA; ^5^ Department of Otolaryngology University of Tennessee Health Science Center Memphis TN USA; ^6^ Department of Pathology and Laboratory Medicine University of Tennessee Health Science Center Memphis TN USA; ^7^ Department of Surgery University of Tennessee Health Science Center Memphis TN USA; ^8^ Division of Cancer Predisposition St. Jude Children's Research Hospital Memphis TN USA

**Keywords:** *DICER1*, multinodular thyroid goiter, pleuropulmonary blastoma

## Abstract

Pulmonary cysts and neoplasms, especially congenital or occurring at a young age, should be thoroughly investigated. Evaluation for *DICER1* mutations should be performed if there is a family history of this syndrome, the lung cyst/neoplasm is a pleuropulmonary blastoma, or other clinical manifestations of this syndrome are present or develop.

## Graphical abstract

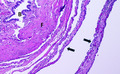

## 1 INTRODUCTION

Pleuropulmonary blastomas are rare malignant tumors that are typically diagnosed prior to the age of 6 years. Germline mutations in the *DICER1* gene predispose to development of pleuropulmonary blastoma and lung cysts, as well as ovarian, thyroid, renal, and other tumors. We report a 16‐year‐old woman, with no prior cancer history, whose diagnosis of a thyroid goiter, and a pleuropulmonary blastoma were sentinel lesions to underlying DICER1 syndrome.

The *DICER1* gene, present on the long arm of chromosome 14, encodes an endoribonuclease involved in the production of mature microRNAs which regulates gene expression through several mechanisms.^1^ *DICER1* pathogenic variants predispose carriers to a rare cancer syndrome, the DICER1 syndrome,^1^ which is inherited in an autosomal dominant manner with decreased penetrance.^2^, ^3^ This syndrome leads to the development of tumors in the lungs, thyroid gland, female reproductive tract, kidneys, small intestines, and the central nervous systerm^3^, ^4^ Pleuropulmonary blastoma (PPB) is the most frequent lesion seen in this syndrome. Thyroid lesions/cancers are also a common finding.^1^ We present the case of a 16‐year‐old woman who presented with a lung cyst and a multinodular thyroid goiter. These findings were key to diagnosis of DICER1 syndrome.

## 2 CASE REPORT

Informed consent was obtained from the mother and patient.

**FIGURE 1:**
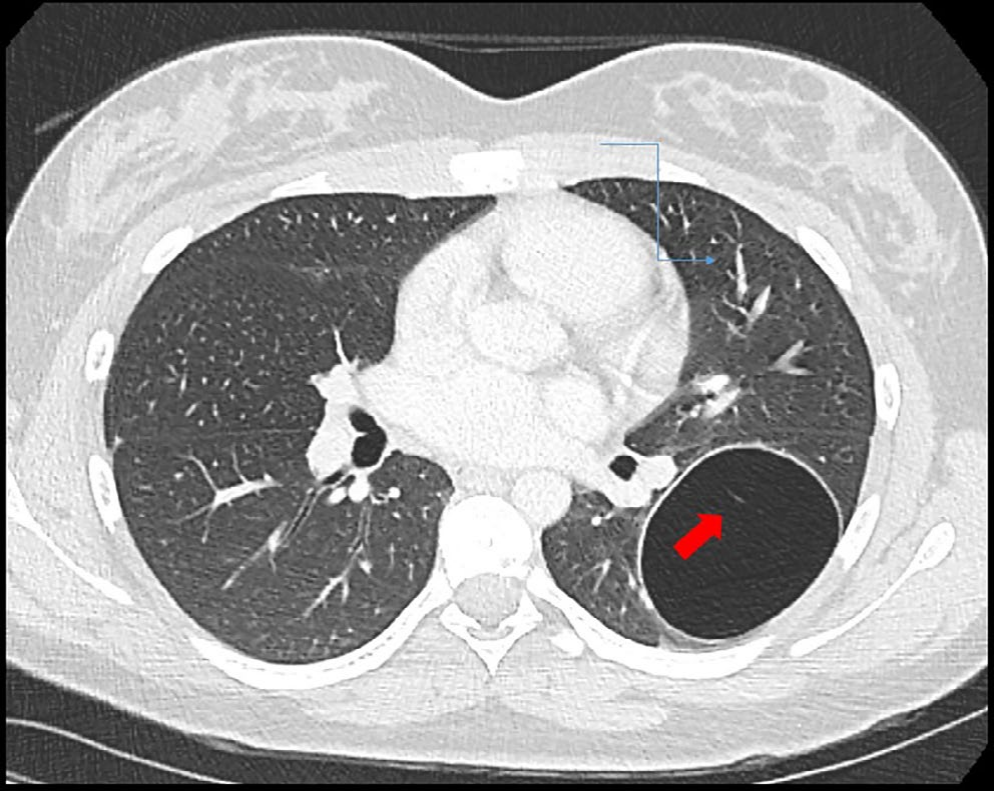
Axial lung window CT image shows an air‐filled cystic lesion within the left lower lobe. The lesion demonstrates a slightly thick wall and a subtle internal linear septation (arrow)

A 16‐year‐old woman was admitted to a Memphis hospital with a 2‐week history of right and left upper quadrant abdominal pain. An ultrasound of the abdomen revealed the presence of a hemoperitoneum. A preoperative chest radiograph (CXR) revealed the presence of a 5 cm × 5 cm × 5 cm cystic lesion in the superior segment of the left lower lobe (LLL). At surgery, the patient was found to have a collapsed ruptured hemorrhagic left ovarian cyst. A left salpingoophrectomy was performed. The pathology reviewed by community and St. Jude Children's Research Hospital (SJCRH) pathologists was benign and consistent with hemorrhagic corpus luteum cyst and cystic follicles. Upon postoperative follow‐up with her gynecologist, a computerized tomography (CT) of the chest was performed to further evaluate the lung cyst. The CT revealed the presence of a 7 cm × 5.5 cm × 6.5 cm unilocular cyst in the superior segment of the LLL (Figure 1) and the presence of a multinodular thyroid goiter.

The patient was referred to the pediatric pulmonology clinic (attending James D. Tutor, MD) by her gynecologist for evaluation of the enlarging cyst in her left lung. The cyst was first noted on a CXR performed at a US military base clinic at age 9 years. The cyst was not further evaluated then. She gave a history of 2‐3 weeks of intermittent left lateral chest pain and an 8‐month history of dysphagia. The physical examination was remarkable only for the presence of a multinodular thyroid goiter. Spirometry was normal. She was referred to the pediatric endocrinology clinic for evaluation of her goiter. A barium esophagram revealed focal narrowing proximal to the thoracic inlet at the level of the thyroid gland. Thyroid function studies were normal, and no thyroid antibodies were detected by the endocrinologist (Hiba Al Zubeidi, M.D.). Given the presence of multinodular thyroid goiter and a cystic lung lesion, the diagnosis of underlying *DICER1* mutation was proffered (by Stephen F. Miller, MD) and she was referred for preoperative cancer predisposition evaluation at SJCRH. Her available family history was notable for a paternal first cousin with unspecified leg tumor diagnosed at age 10 years; maternal grandmother and maternal grandmother's sister were both reported to have breast cancer diagnosed in their thirties. No prior genetic testing was known to have been performed in family members.

Clinical germline testing for genes associated with personal and family history revealed a heterozygous pathogenic variant in exon 18 of the *DICER1* gene (p.Asp940Ter). While this variant is not present in population databases nor reported in the *DICER1* literature, it is anticipated to result in absent or disrupted protein product due to creation of a premature translational stop signal at amino acid 940. These results are consistent with a diagnosis of DICER1 syndrome in our patient. No other variants were identified in other genes tested (see Methods section). Subsequent *DICER1* germline testing of our patient's mother and father was negative for *DICER1* pathogenic variant, suggesting that this variant is a de novo occurrence in our patient. *DICER1* germline testing was offered to our patient's half‐siblings due to the possibility of germline mosaicism in one parent (patient does not have full‐siblings). To date, both maternal half‐siblings tested negative. Her paternal half‐siblings have not yet proceeded with testing.

**FIGURE 2:**
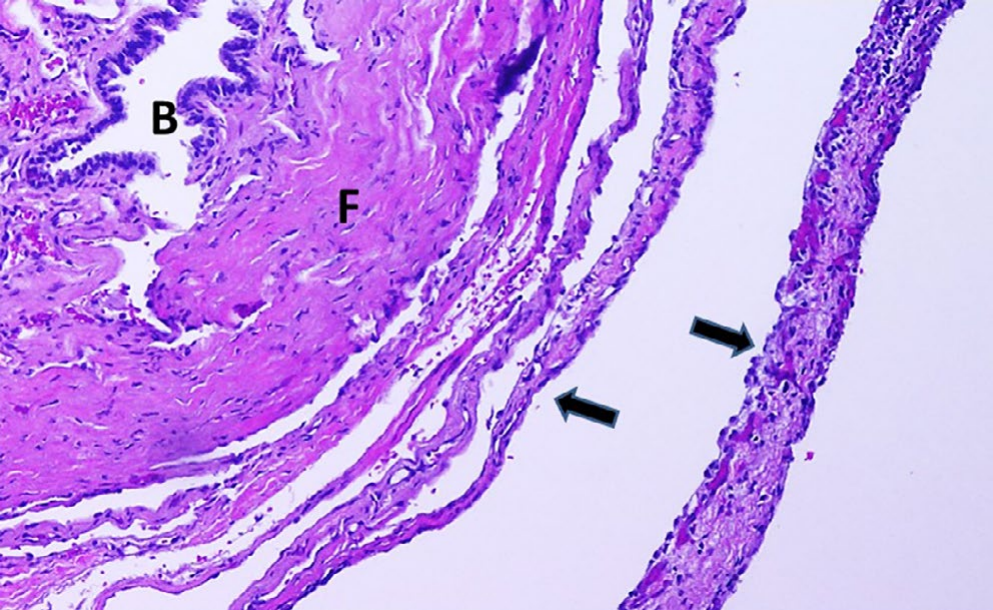
Pleuropulmonary blastoma type 1r (type 1‐regressed PPB). Hematoxylin and Eosin (HE) stain, ×100. B: bronchiole; F: fibrous cyst wall; Arrow: cystic septa

The left lobe of the thyroid was removed by the otolaryngologist (Anthony Sheyn, MD), and the LLL was removed thoracoscopically by the pediatric surgeon (Regan Williams, MD). The pathology of the lung cyst revealed a PPB, type 1‐regressed (Figure 2), and the pathology of the thyroid tissue was follicular adenomas with a background of adenomatoid hyperplasia (both interpreted by Jie Zhang, MD).

She has completely recovered from the surgery. Thyroid function is normal. Baseline tumor surveillance for DICER1 syndrome which included renal and pelvic ultrasound was normal and long‐term follow‐up with the cancer predisposition team is planned. She and her family members were counseled about DICER1 syndrome (by Rose B. McGee, MS, CGC), and she has completed successful oocyte cryopreservation procedure with SJCRH fertility clinic given tumor risk to her remaining right ovary.

## 3 METHODS

The patient's peripheral blood sample was sent to Invitae Corporation laboratory for clinical genetic testing to detect sequence changes and deletion/duplications in the following genes: *CDC73*, *DICER1*, *FLCN*, *PTCH1*, *TP53*, and *VHL*. Per the Methods section of the patient's clinical test report, genomic DNA was extracted and next‐generation sequencing (NGS) was performed on an Illumina platform with >50× depth or supplemented with additional analysis. The sequence reads were aligned to (GRCh 37) reference sequence, and variants were identified using clinically relevant transcripts for each gene. Orthogonal technologies (eg, Sanger sequencing, Pacific Biosciences SMRT sequencing) were used to confirm clinically significant findings not meeting stringent NGS quality metrics. The *DICER1* pathogenic variant was the only variant reported on our patient's test report. Parental and sibling testing was submitted to Invitae for NGS familial testing for *DICER1* gene to detect the presence or absence of our patient's identified pathogenic variant. Similar sequencing methods as above were used by the laboratory. Neither the patient's *DICER1* pathogenic variant nor any other *DICER1* pathogenic/likely pathogenic variants were detected in either parent's sample.

## 4 DISCUSSION

Pleuropulmonary blastomas are malignant tumors that arise from the primitive interstitial mesenchyme during lung development. They are generally aggressive with high rates of metastasis to the liver, brain, and spinal cord.^5^ There are three types of PPB. Type 1 is a purely cystic lesion. It is usually diagnosed by 10 months of age^5^ and has a survival rate of 91%.^3^ Purely cystic tumors that lack a primitive cell component, as in our patient, are classified as type 1r, which signifies regression or nonprogression to more malignant types.^6^ Type 2, a combination cystic and solid lesion, usually presents by 34 months of age^5^ and has a survival rate of 71%.^3^ Type 3, a purely solid lesion, usually presents by 44 months of age^5^ and has a survival rate of 53%.^3^ The patient's lesion was first reported on a CXR when she was 9 years old but it was not further evaluated at that time. The 2018 international consensus guidelines for DICER1 syndrome recommend *DICER1* germline testing for an individual with childhood lung cyst(s), especially if the cyst(s) are multiseptated or bilateral.^3^

Treatment of cystic PPBs includes surgical excision with or without adjuvant chemotherapy and radiation therapy.^3^ Since the pathology of our patient's lung lesion was type 1 regressed PPB, only surgical resection of the LLL of the lung was indicated. Surveillance for PPB and lung cysts in the setting of DICER1 syndrome includes chest imaging with varying modalities (primarily CXRs) and frequency, depending on the patient's age.^3^

The presence of a lung cyst and a multinodular thyroid goiter in our patient was critical to the ultimate identification of underlying DICER1 syndrome with corresponding implications to her clinical and reproductive management and to her family members. An extensive pedigree and genetic evaluation of the patient's immediate family members revealed no other family members with the DICER1 syndrome indicating a presumed de novo mutation in the patient. The patient will continue to have annual physical examination with a targeted review of systems and DICER1 syndrome imaging studies with type and frequency based on age, and presence or absence of critical symptoms as per recent consensus guidelines.^3^ The identification of DICER1 syndrome also provides reproductive options for family planning and implications for prenatal management. At‐risk fetuses should be monitored for the prenatal development of lung cysts.^3^

## CONFLICT OF INTEREST

None declared.

## AUTHOR CONTRIBUTIONS

JDT: MD, is pediatric pulmonologist who treated the patient, wrote, and revised the manuscript, found some of the references, and serves as the corresponding author. SFM: MD, the radiologist: provided and labeled Figure 1 and provided recommendations for revising the manuscript. HAZ: MD, is endocrinologist who treated the patient and reviewed the manuscript. AS: MD, is otolaryngologist who operated on the patient's thyroid gland and reviewed the manuscript. JZ: MD, is pathologist who reviewed the tissue from the lung, provided, and labeled Figure 2 and provided recommendations for revising the manuscript. RW: MD, is surgeon who removed the patient's lung lobe and reviewed the manuscript. RM: MS, CGC: is genetics expert who worked with the patient and family, helped revise the whole manuscript, especially the Methods section, and found some of the main references for the paper.
